# Chemotherapy-induced pyroptosis is mediated by BAK/BAX-caspase-3-GSDME pathway and inhibited by 2-bromopalmitate

**DOI:** 10.1038/s41419-020-2476-2

**Published:** 2020-04-24

**Authors:** Lei Hu, Meng Chen, Xueran Chen, Chenggang Zhao, Zhiyou Fang, Hongzhi Wang, Haiming Dai

**Affiliations:** 1 Anhui Province Key Laboratory of Medical Physics and Technology, Center of Medical Physics and Technology, Hefei Institutes of Physical Science, Chinese Academy of Sciences, 230031 Hefei, China; 2 University of Science and Technology of China, 230026 Hefei, China; 3 Hefei Cancer Hospital, Chinese Academy of Sciences, 230031 Hefei, China

**Keywords:** Cell death, Cell signalling

## Abstract

Many chemotherapy treatments induce apoptosis or pyroptosis through BAK/BAX-dependent mitochondrial pathway. BAK/BAX activation causes the mitochondrial outer membrane permeabilization (MOMP), which induces the activation of pro-apoptotic caspase cascade. GSDME cleavage by the pro-apoptotic caspases determines whether chemotherapy drug treatments induce apoptosis or pyroptosis, however, its regulation mechanisms are not clear. In this study, we showed that TNFα+CHX and navitoclax-induced cancer cell pyroptosis through a BAK/BAX-caspase-3-GSDME signaling pathway. GSDME knockdown inhibited the pyroptosis, suggesting the essential role of GSDME in this process. Interestingly, GSDME was found to be palmitoylated on its C-terminal (GSDME-C) during chemotherapy-induced pyroptosis, while 2-bromopalmitate (2-BP) could inhibit the GSDME-C palmitoylation and chemotherapy-induced pyroptosis. Mutation of palmitoylation sites on GSDME also diminished the pyroptosis induced by chemotherapy drugs. Moreover, 2-BP treatment increased the interaction between GSDME-C and GSDME-N, providing a potential mechanism of this function. Further studies indicated several ZDHHC proteins including ZDHHC-2,7,11,15 could interact with and palmitoylate GSDME. Our findings offered new targets to achieve the transformation between chemotherapy-induced pyroptosis and apoptosis.

## Introduction

Pyroptosis is a newly identified programmed cell death (PCD), characterized by cell swelling, forming large bubbles on the plasma membrane and disruption of plasma membrane^1^. Originally regarded as an innate immune mechanism that occurs in macrophages, monocytes, dendritic cells (DCs), and T cells upon the stimulations by pathogens or the products from pathogens^2,3^, pyroptosis was found to be mediated by the pro-inflammatory caspases^4,5^. More recently, pyroptosis was also found to be mediated by pro-apoptotic caspases in cancer cells and human primary cells after induced by chemotherapies^6,7^.

As a pro-inflammatory form of PCD, pyroptosis can protect multicellular organisms from invading pathogenic bacteria and microbial infections. However, its overactivation will lead to sepsis and lethal septic shock^8^. Pro-inflammatory caspases that induce pyroptosis include caspase-1 in both human and mice, caspase-4 and -5 in human, and caspase-11 in mice. Caspase-1 mediated pyroptosis is often activated by cannonical inflammasomes^9^, while caspase-4/5/11 mediated pyroptosis is often activated within non-canonical inflammasomes^10^.

Pyroptosis can also be induced by pro-apoptotic caspases, such as caspase-3^6,7^ and caspase-8^11,12^. Caspase-3 is often activated by the death receptor mediated apoptotic pathway through the activation of caspase-8 or the mitochondrial apoptotic pathway through the activation of caspase-9^13^. Many anti-cancer drugs induced cancer cell death through the mitochondrial pathway, which was controlled by BCL2 family proteins. Two BCL2 family members, BAK and BAX can form pores on the mitochondrial outer membrane (MOM) after activation, resulting in the release of mitochondrial inter-membrane components, which will cause the activation of caspase cascade^14–16^.

The cleavage of gasdermin family proteins GSDMD and GSDME mediate pyroptotic characteristics. GSDMD is cleaved by activated pro-inflammatory caspases after Asp275 (Asp276 in mouse), generating an N-terminal domain (GSDMD-N)^17–19^. Originally defined as a putative oncosuppressor protein^20^, GSDME, is cleaved by caspase-3 after Asp270 generating an N-terminal GSDME (GSDME-N) upon the treatment of anti-cancer agents^6,7,21^. Both GSDMD-N and GSDME-N oligomerize and form large pores on the plasma membrane to induce plama membrane disruption, leading to the release of cellular contents, including pro-inflammatory mediators, and alamins. While caspase-3 is a pro-apoptotic caspase, the cleavage of GSDME by activated caspase-3 after chemotherapy drug treatments provides a cross-talk between the pro-infammatory pryroptosis and anti-inflammatory apoptosis.

GSDME expression levels vary in different cell types and tissues. High levels of GSDME lead to pyroptosis while cells with low levels undergo apoptosis upon chemotherapy treatments^9^. In the presence of GSDME, chemotherapy drug-induced caspase-3 activation often leads to a pyroptotic characteristics^6,7^, because pyroptosis progresses faster than apoptosis^18^. Although GSDME cleaved by pro-apoptotic caspases convert apoptosis to pyroptosis, however, its regulation mechanisms are not clear.

As one of the most common posttranslational modifications of proteins, palmitoylation is defined by the addition of saturated 16-carbon palmitic acid to specific cysteine residues^22^ and plays an important role in multiple intracellular physiological processes^23–26^. 2-BP is one of the most commonly used palmitoylation inhibitors, which can directly and irreversibly inhibit the palmitoyltransferase activity of all DHHC (Asp-His-His-Cys) proteins^27^. In this study, we not only found that chemotherapy-induced pyroptosis was mediated by the BAK/BAX-caspase-3-GSDME pathway, but also showed either BAK or BAX alone can mediate this process. More importantly, we found that GSDME was palmitoylated during chemotherapy-induced pyroptosis, while 2-BP could inhibit this process.

## Results

### BAK and BAX enable TNFα+CHX and navitoclax to induce pyroptosis

**Fig. 1:**
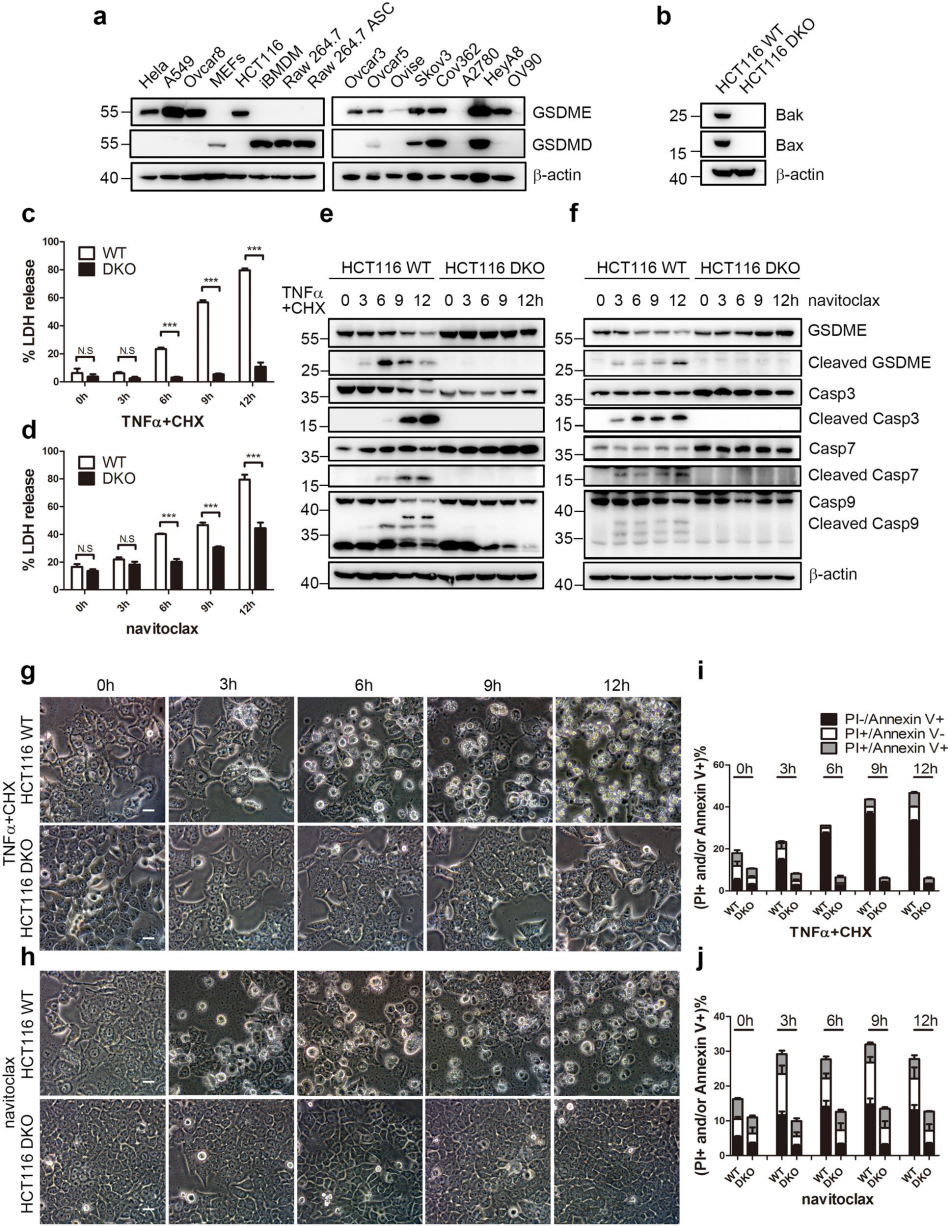
BAK/BAX deletion inhibits TNFα+CHX or navitoclax-induced pyroptosis. **a** Expresssion level of GSDMD and GSDME in various cell lines. **b** Expression of BAK/BAX in wild type (WT) and *BAK/BAX* double knockout (DKO) HCT116 cells. **c**, **d** At the indicated time points, the percentage of LDH release in the culture supernatants from HCT116 WT and DKO was measured after TNFα+CHX (**c**) or navitoclax (**d**) treatment. Error bars in this and subsequent figures: mean ± SD of three independent experiments. *P < 0.05; **P < 0.01; ***P < 0.001. **e**, **f** At the indicated time points, immunoblottings of GSDME, caspases, and cleaved caspases were performed in HCT116 WT and DKO cells treated with TNFα+CHX (**e**) or navitoclax (**f**). **g**, **h** After HCT116 WT and DKO cells were treated with TNFα+CHX (**g**) or navitoclax (**h**) for indicated time, representative microscopic images were taken. Scale bar in this and subsequent figures, 100 μm. **i**, **j** After TNFα+CHX (**i**) or navitoclax (**j**) treatment, HCT116 cells were collected at the indicated time points and stained with FITC-Annexin V and PI. The percentages of single PI positive, single FITC-Annexin V positive, and FITC-Annexin V/PI double positive HCT116 (WT or DKO) were detected by flow cytometry.

Previous studies have shown chemotherapy drugs induce pyroptosis through the GSDME cleavage^6,7^, while bacteria or LPS induces pyroptosis through GSDMD cleavage^17–19^. Many cancer cells do not express GSDME due to its promoter methylation^28–30^. We first screened several cancer cell lines for the expression of GSDMD and GSDME (Fig. 1a and Supplementary Fig. 1a). The colorectal cancer cell line HCT116 was chosen because it expresses high levels of GSDME but not GSDMD (Fig. 1a).

Many anti-cancer drugs induce apoptosis through the BAK/BAX-dependent apoptosis pathway^31,32^, however, whether this pyroptosis was dependent on BAK/BAX is not clear. To study this, wild type (WT) and BAK^−/−^BAX^−/−^ (DKO) HCT116 cells (Fig. 1b) were first treated with navitoclax, the BCL2, BCLxL, and BCLw inhibitor^33,34^. After HCT116 cells were exposed to navitoclax (Supplementary Fig. 1b–d), we found navitoclax induced a concentration-dependent GSDME cleavage and also caspase-3, 7, and 9 cleavages (Supplementary Fig. 1b). Consistent with these observations, we observed the LDH release in WT HCT116 cells (Supplementary Fig. 1c) and pyroptotic morphologies, indicated by cell swelling and accompanied by large bubbles blown from the plasma membrane (Supplementary Fig. 1d). These observations together confirmed pyroptosis was induced in WT HCT116 cells upon navitoclax treatment.

A time-course study of HCT116 cells treated with TNFα+CHX and navitoclax was further performed (Fig. 1 and Supplementary Fig. 2). Both treatments induced a time-dependent pyrotosis in WT HCT116 cells, as indicated by the LDH release (Fig. 1c, d), the cleavage of GSDME (Fig. 1e, f), the observation of pyroptotic cell morphologies (Fig. 1g, h) and the increased percentages of Annexin V+ and/or PI+ cells (Fig. 1i, j, Table S1 and Supplementary Fig. 2).

In contrast to HCT116 WT cells, when DKO HCT116 cells were treated, both concentration-dependent and time-dependent pyroptosis were inhibited significantly, as indicated by the decrease of the percentage of LDH release (Fig. 1c, d and Supplementary Fig. 1c), the GSDME cleavage (Fig. 1e, f and Supplementary Fig 1b), the percentage of pyroptotic cell morphology (Fig. 1g, h), and the percentage of Annexin V+ and/or PI+ cells (Fig. 1i, j, Table S1 and Supplementary Fig 2). While GSDME has been suggested to be cleaved by activated caspase-3^6,7^, we also observed the diminish of cleaved caspase-3, 7, and 9 in DKO HCT116 cells compared to WT (Fig. 1e, f and Supplementary 1b), in agreement with previous studies^35^.

We also tested these drugs in two other cell lines Hela and HeyA8 (Supplementary Figs. 3 and 4), which also express high levels of GSDME. TNFα+CHX and navitoclax - induced time-dependent LDH release (Supplementary Fig. 3a, b) and pyroptotic cell morphorlogies in Hela cells (Supplementary Fig. 3c). Moreover, actinomycin D also induced Hela cell pyroptosis (Supplementary Fig. 4a, c). In HeyA8, which expressed both GSDME and GSDMD (Fig. 1a), TNFα+CHX induced a time-dependent LDH release and pyroptotic morphology (Supplementary Fig. 4b, d). These results suggested that anti-cancer drugs induce pyroptosis in multiple cancer cell lines.

Among the above treatments, TNFα-induced caspase-3 activation could either directly induced by activated caspase-8 in Type I cells, or induced by the mitochondria pathway through BID cleavage in type II cells^36–38^. HCT116 has been previously reported to be a type II cell because of the low expression of cell surface death receptors^39,40^. Consistent with this, BID knockdown attenuated the GSDME cleavage induced by TNFα+CHX but not navitoclax (Supplementary Fig. 5), further supporting the idea that BAK/BAX activation plays an essential role in chemotherapy-induced pyroptosis.

### Pyroptosis can be mediated by sole BAK or BAX

**Fig. 2:**
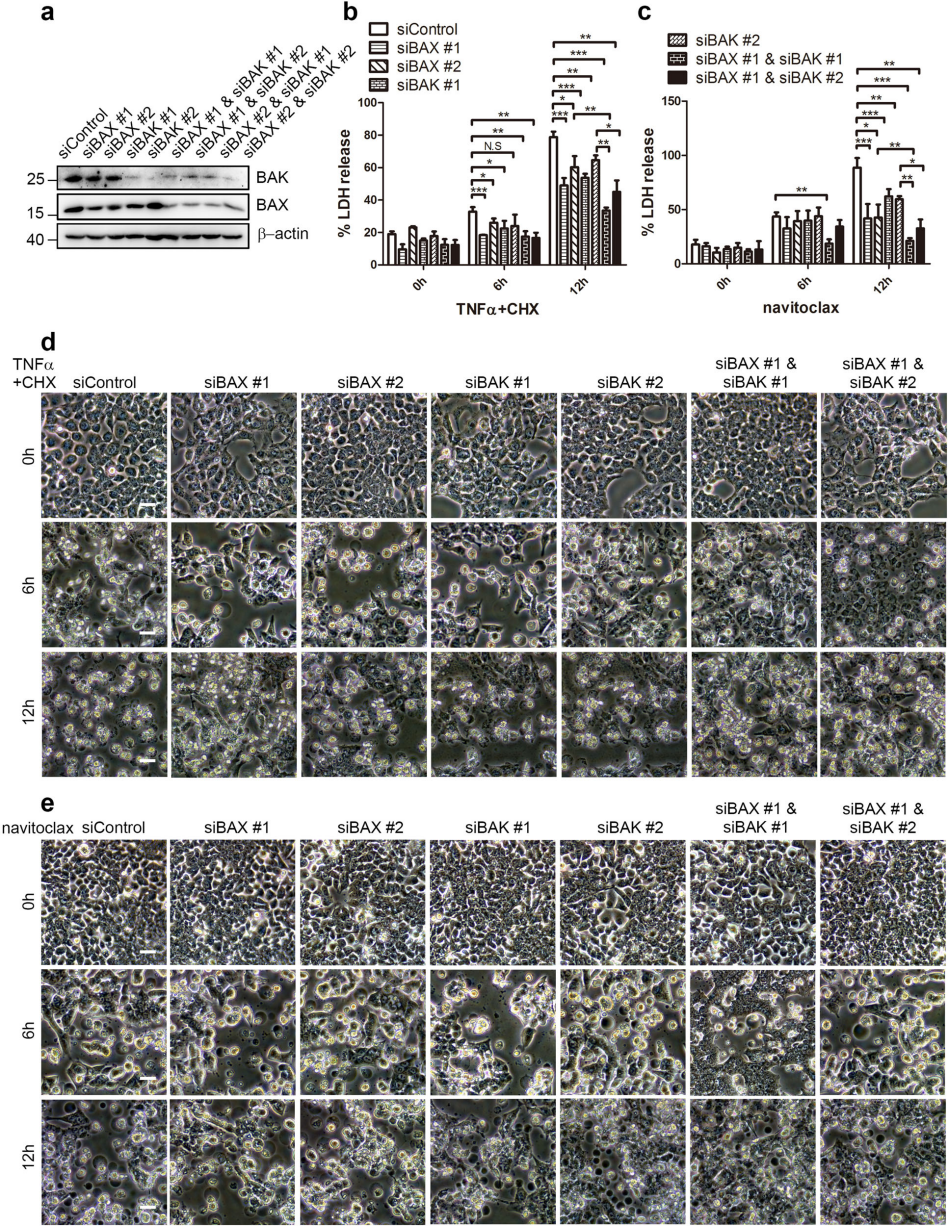
Either BAK or BAX knockdown decreases TNFα+CHX or navitoclax-induced pyroptosis. **a** Efficiency of BAK and/or BAX knockdown was detected by immunoblotting. **b–e** After BAK siRNA, BAX siRNA, BAK siRNA+BAX siRNA, or negative control siRNA were transfected into HCT116 cells by Lipofectamine RNAiMAX, cells were reseeded into 24-well plate followed by treatment of TNFα+CHX (**b**, **d**) or navitoclax (**c**, **e**) for 24 h. Culture supernatants were collected to measure the percentage of LDH release (**b**, **c**) and phase contrast images were taken (**d**, **e**) at the indicated time points.

To further assess the contribution of BAK and BAX in GSDME-dependent pyroptosis, BAK or/and BAX were knocked down by siRNAs (Fig. 2a). As shown in Fig. 2b, the LDH released by TNFα+CHX was signicantly inhibited by the knock-down of either BAK or BAX (except siBAK #2 at 6 hour). Moreover, the LDH release by navitoclax was also decreased at 12 h by all the siRNAs, suggesting that both BAK and BAX contributed to the LDH release (Fig. 2c). In addition, both the single BAK and BAX knockdowns showed more LDH release than the BAK/BAX double knockdowns (Fig. 2b, c), again arguing that either BAK or BAX had the ability to induce LDH release. The phase-contrast images of morphology further supported the above results (Fig. 2d, e). Consistent with these observations, caspase-3, 7, 9 and the GSDME cleavage also decreased remarkably after BAK or BAX siRNA treatments (Supplementary Fig. 6). These observations aligned the view that BAK or BAX alone could mediate chemotherapy-induced pyroptosis.

### Caspase-3 activity is required in BAK/BAX-mediated pyroptosis

**Fig. 3:**
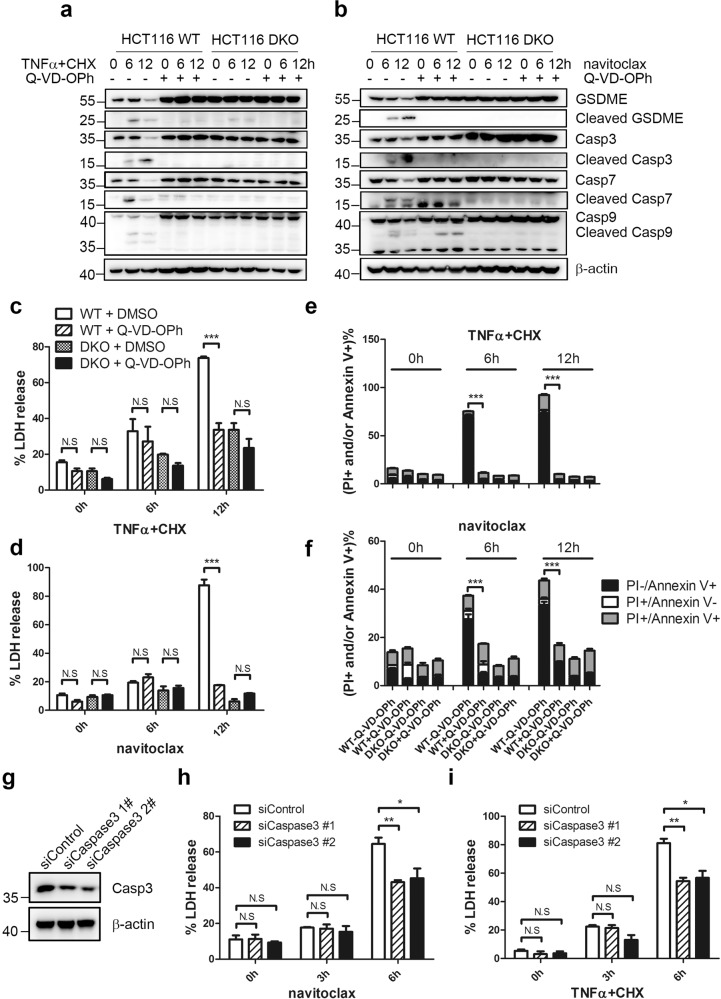
Caspase-3 is required for TNFα+CHX and navitoclax-induced pyroptosis. **a**, **b** Immunoblottings of GSDME, caspases, and cleaved caspases in HCT116 WT and DKO cells treated with TNFα+CHX (**a**) or navitoclax (**b**) in the absence or presence of Q-VD-OPh at the indicated time points were performed. **c**, **d** The percentage of LDH release in the culture supernatants from HCT116 WT and DKO was measured after TNFα+CHX (**c**) or navitoclax (**d**) treatments in the absence or presence of Q-VD-OPh at the indicated time points. **e**, **f** After HCT116 WT and DKO cells were treated with TNFα+CHX (**e**) or navitoclax (**f**) in the absence or presence of Q-VD-OPh, the percentages of single PI positive, single FITC-Annexin V positive, and FITC-Annexin V/PI double positive cells were detected by flow cytometry at the indicated time points. **g** Efficiency of caspase-3 knockdown was detected by immunoblotting. **h**, **i** After HCT116 WT cells were transfected with caspase-3 siRNAs followed by TNFα+CHX (**h**) or navitoclax (**i**) treatments, culture supernatants were collected to measure the percentage of LDH release.

Activated caspase-3 have been reported to cleave GSDME to induce pyroptosis^6,7^. Moreover, caspase-9 and subsequent caspase-3, 7 are all activated after chemotherapy-induced BAK/BAX activation^41,42^. To identify the caspase(s) responsible for GSDME cleavage, the pan-caspase inhibitor Q-VD-OPh was first used. Pre-incubation of Q-VD-OPh not only abolished TNFα+CHX or navitoclax-induced caspase-3, 7, 9 and GSDME cleavage (Fig. 3a, b), but also diminished the LDH release in WT but not in DKO HCT116 cells at 12 h (Fig. 3c, d). The morphological changes also supported the above results (Supplementary Fig. 7a, b). Moreover, the percentage of Annxin V positive cells (but not PI positive or PI/Annexin V double positive cells) decreased significantly in the WT group after Q-VD-OPh pre-treated compared to the controls (Fig. 3e, f and Table S2). To further specify if caspase-3 is critical, we knocked down caspase-3 in HCT116 cells (Fig. 3g). Indeed, knockdown of caspase-3 by two different siRNAs significantly reduced the LDH release induced by TNFα+CHX and navitoclax at 6 h (Fig. 3h, i). Moreover, pre-treatment of the caspase-3 specific inhibitor Q-DEVD-OPh treatment also significantly reduced the LDH release induced by TNFα+CHX and navitoclax (Supplementary Fig. 7c, d). Taken together, the results suggested caspase-3 was required for TNFα+CHX and navitoclax-induced pyroptosis.

### GSDME is crucial in anti-cancer drug-induced pyroptosis

**Fig. 4:**
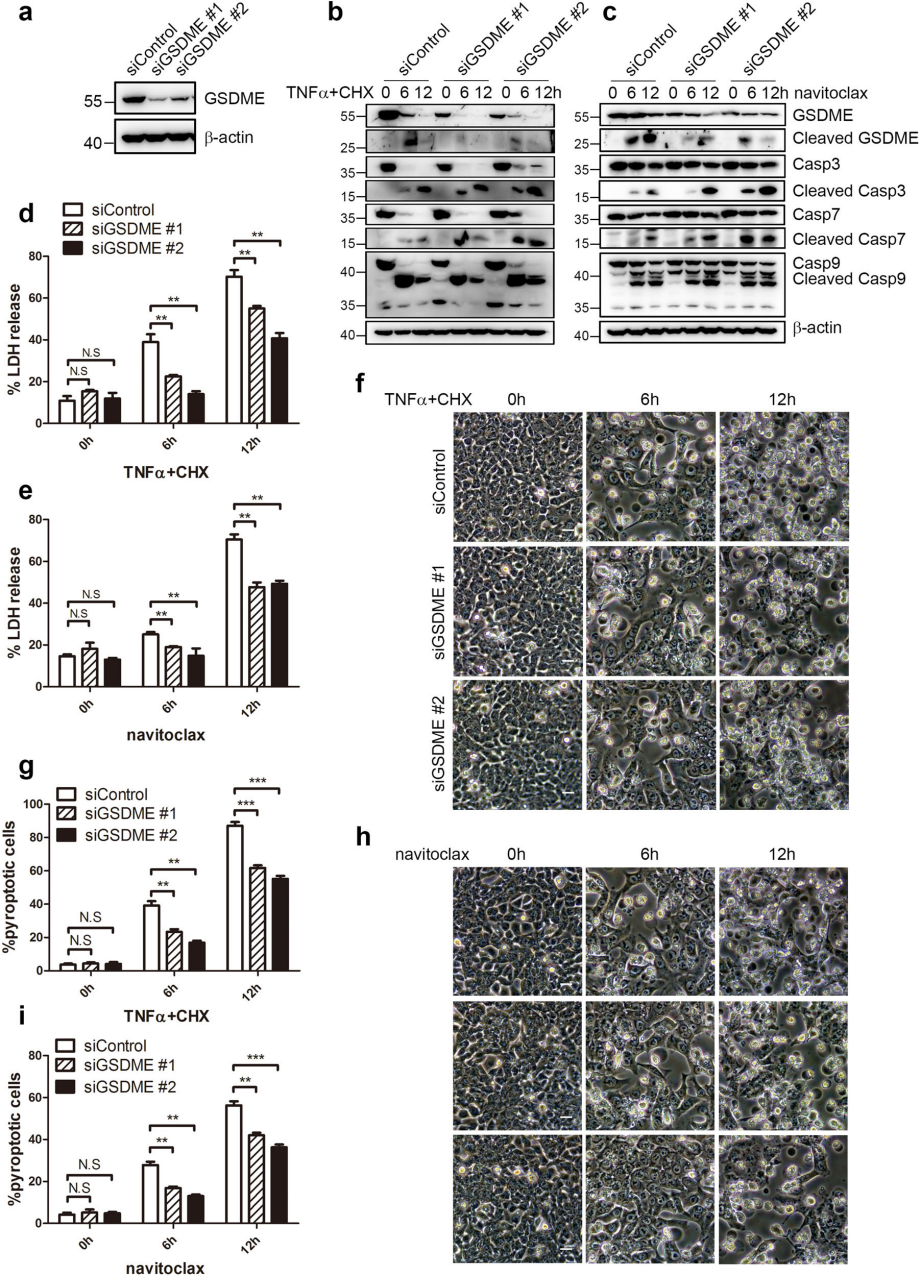
GSDME knockdown decreases pyroptosis induced by TNFα+CHX or navitoclax. **a** Efficiency of GSDME knockdown was detected by immunoblotting. **b**–**e** After HCT116 cells were transfected with GSDME siRNAs and negative control, cells were reseeded and treated with TNFα + CHX (**b, d**) or navitoclax (**c, e**) for the indicated time. The cells were collected for immunoblotting analysis (**b**, **c**) and culture supernatants were collected to detect the percentage of LDH release (**d**, **e**). **f**–**i** After HCT116 cells were transfected with GSDME siRNAs and negative control siRNA, cells were reseeded and treated with TNFα+CHX (**f**, **g**) or navitoclax (**h**, **i**). Phase contrast images were taken at the indicated time points (**f**, **g**) and the percentage of pyroptotic cells was calculated (**h**, **i**).

Further, two siRNAs targeting GSDME were applied. While more than 50% of GSDME was knocked down by either siRNA (Fig. 4a), the knockdown cells displayed lower levels of both uncleaved and cleaved GSDME after TNFα+CHX or navitoclax treatments (Fig. 4b, c). Moreover, the caspase-3, 7, and 9 cleavage were not decreased and even increased compared to the control cells (Fig. 4b, c), in agreement with the idea that GSDME cleavage was downstream of caspases activations^6,7^. More caspase-3, 7, and 9 cleavages were observed after GSDME knockdown, which might because more living cells with caspase activations were harvested when pyroptosis was blocked. Furthermore, TNFα+CHX and navitoclax-induced LDH releases were significantly reduced in GSDME knockdown cells (Fig. 4d, e), suggesting that the LDH release was downstream of GSDME cleavage^6,7^. In addition, cells with pyroptotic morphology also decreased apparently after GSDME knockdown (Fig. 4f–i). Taken together, these results suggested that GSDME played a key role in BAK/BAX-caspase-3 mediated pyroptosis induced by TNFα+CHX and navitoclax.

### GSDME is modified during anti-cancer drug-induced pyroptosis

**Fig. 5:**
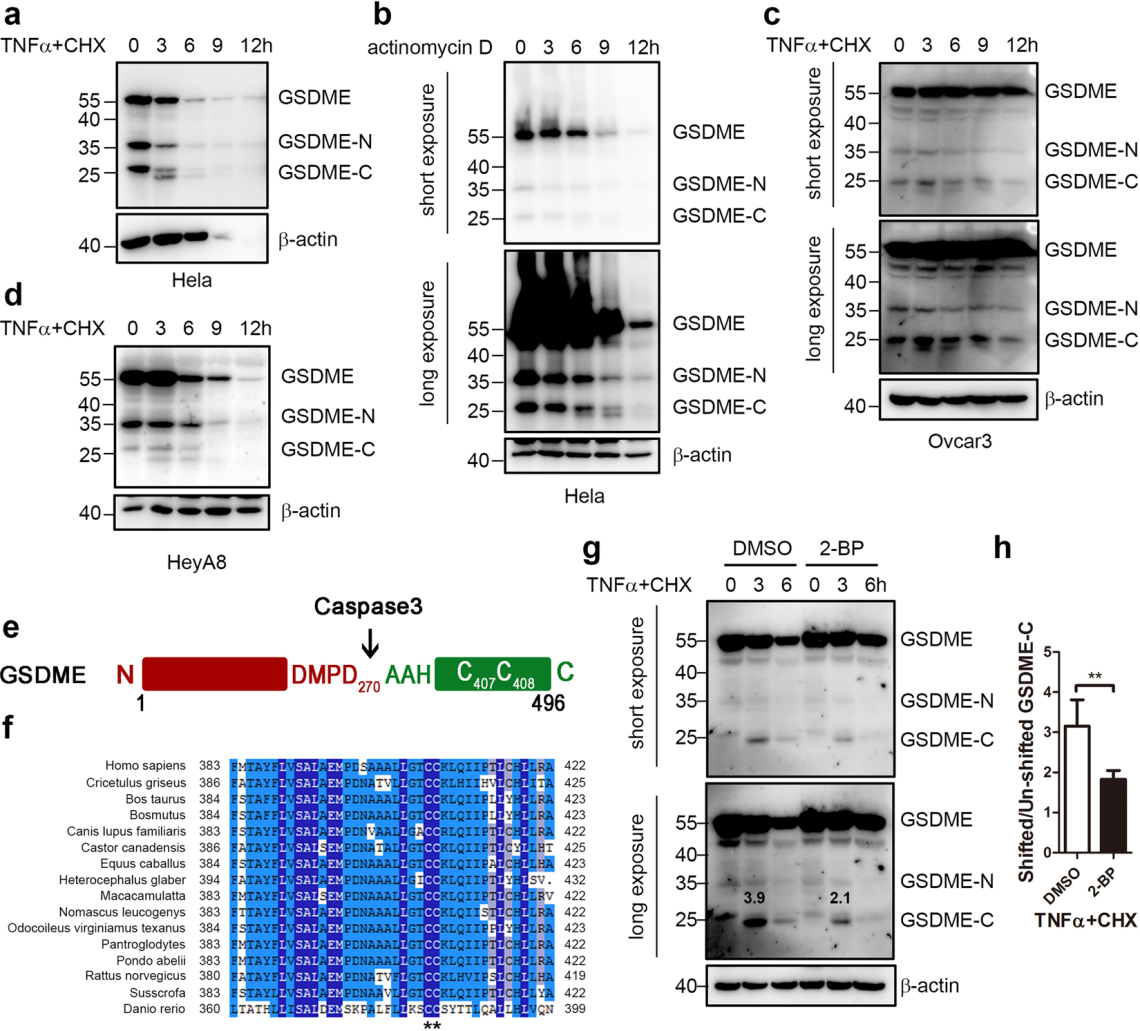
GSDME is modified during anti-cancer drug-induced pyroptosis. **a**, **b** After Hela cells were treated with TNFα+CHX (**a**) or actinomycin D (**b**) for the indicated time, the cells were subjected to immunoblotting. **c**, **d** TNFα+CHX treated Ovcar3 and HeyA8 cells for the indicated time and then harvested for immunoblotting. **e** The potential palmitoylation sites were predicted by CSS-Palm 4.0. **f** Sequence alignment of the potential palmitoylation site of GSDME from Danio rerio to Homo sapiens. Asterisk represents the conserved Cysteine residues were highlighted. **g**, **h** After Hela cells were pre-incubated with broad-spectrum palmitoylation inhibitors 2-BP followed by the treatment of TNFα+CHX for the indicated time, the cells were subjected to immunoblotting (**g**). Numbers indicated the ratio of optical density of shifted GSDME-C band to the unshifted GSDME-C band were obtained from four independent assays by using ImageJ software (**h**).

TNFα+CHX and navitoclax not only induced pyroptosis in HCT116 cells, but also in Hela cells and HeyA8 (Supplementary Figs. 3 and 4). The anti-cancer drugs induced GSDME cleavage was further investigated in these cells (Fig. 5a–d). In Hela cells, a time dependent of GSDME cleavage upon TNFα+CHX treatment was observed (Fig. 5a). However, cleaved GSDME decreased after 6 h treatment, which might be because of the cell death or the further degradation of the GSDME fragments. Interestingly, after TNFα+CHX treatment, not only the degradation of GSDME, but a shift of the GSDME-C band at 3 h was also found, suggesting a potential modification. This shifted band of GSDME-C was also observed upon other chemotherapy treatments or in other cell lines, such as in Hela cells treated with actinomycin D, or in HeyA8 and Ovcar3 cells treated with TNFα+CHX (Fig. 5b–d). Knock-down of GSDME with two different siRNAs confirmed that both bands of GSDME-C were specific (Supplementary Fig. 8a). Although GSDME-C could be recognized by the GSDME antibody in western blotting (Fig. 5a–d and Supplementary Fig. 8a), it could not be harvested in an immuno-precipitation assay using the same antibody (Supplementary Fig. 8b), limiting our further identification.

GSDME is normally cytosolic, and the inhibitory C-terminal domain is cleaved by activated caspase-3 during pyroptosis, resulting in the translocation of GSDME-N to the plasma membrane^6,7^. While palmitoylation has been predicted to modify the gasdermin family proteins^43^, we speculated that the GSDME modification might be palmitoylation. To test the idea, the potential palmitoylation sites on GSDME was predicted using CSS-Palm 4.0. We first test several known palmitoylated proteins on this program, such as CD9, CLIP3, HRAS, RHOB, GRK6, GAP43, CKAP4, NRAS, and TGS4, all of which indicated positive results and the right palmitoylation sites (Table S4). Further analysis also indicated GSDME could be palmitoylated, while residues C407 and C408 were the targeted sites (Fig. 5e), both of which were conserved from Danio rerio to Homo sapiens (Fig. 5f). Moreover, pre-treatment of 2-BP, the palmitoylation inhibitor, caused a significant decrease of this shifted GSDME-C band (Fig. 5g, h), supporting the idea that the GSDME-C modification was palmitoylation.

### 2-BP inhibited TNFα+CHX induced pyroptosis but not total cell death

**Fig. 6:**
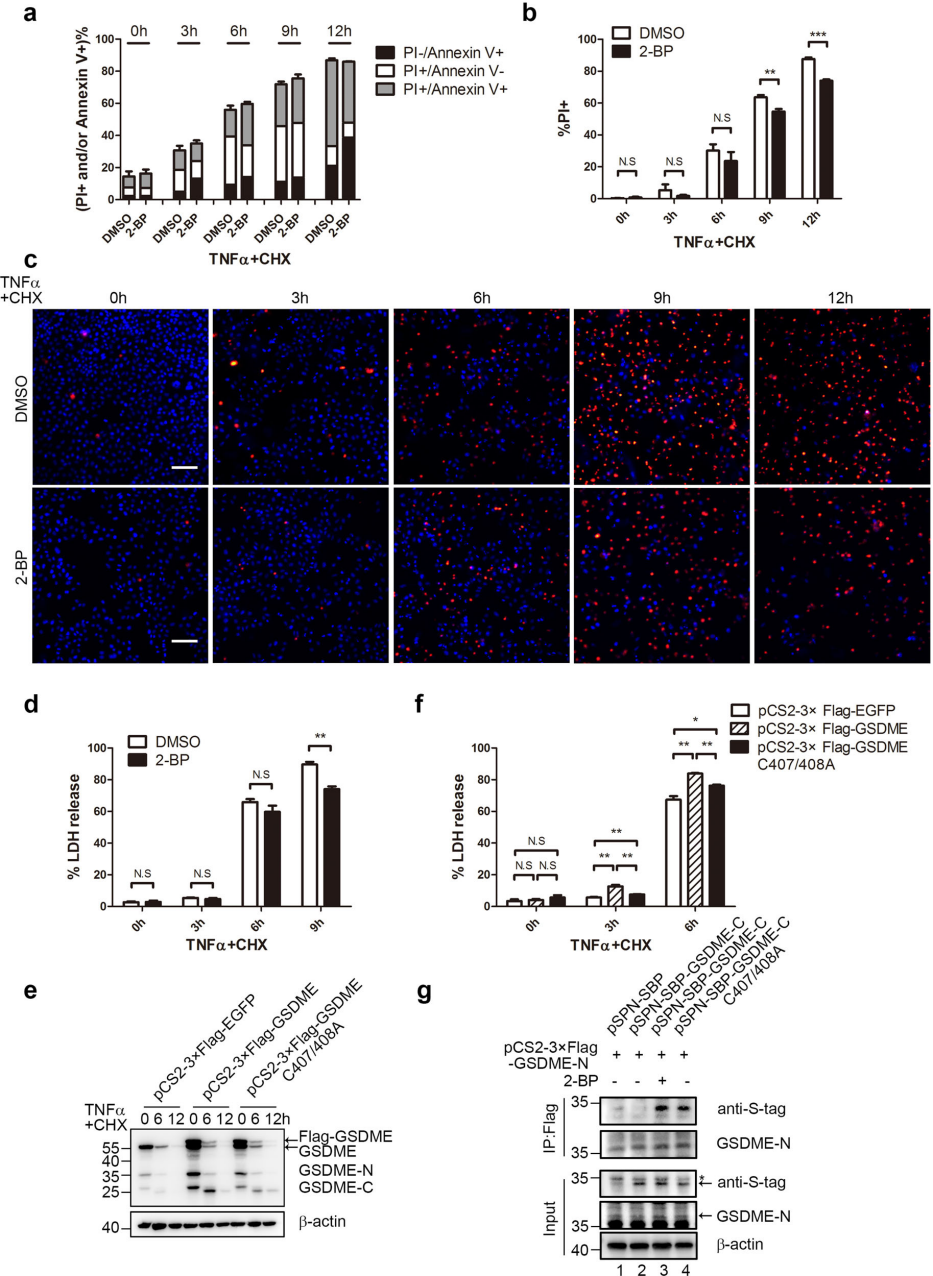
2-BP treatment inhibited TNFα+CHX induced pyroptosis. **a** After 2-BP and TNFα+CHX treatment, Hela cells were collected at the indicated time points and stained with FITC-Annexin V and PI. The percentages of single PI positive, single FITC-Annexin V positive and FITC-Annexin V/PI double positive cells were detected by flow cytometry. **b**, **c** After 2-BP and TNFα+CHX treatment for the indicated time, Hela cells were subjected to Hoechst 33342 and PI double staining. Fluorescent microscopic images were taken at the indicated time points (**c**) and the ratio of PI positive cells were determined by Image J software (**b**). **d** After Hela cells were treated with 2-BP and TNFα+CHX for the indicated time, the culture supernatants were collected to measure the percentage of LDH release. **e**, **f** After Hela cells were transfected with GSDME WT or C407A/C408A mutant followed by the treatment of TNFα+CHX for the indicated time, cells and supernatants were collected for immunoblotting with anti-GSDME and anti-*β*-actin antibodies (**e**) and LDH detection (**f**). **g** 24 h after 3× Flag-tagged GSDME-N and WT or mutated S-tagged GSDME-C were co-transfected, Hela cells were treated with DMSO or 2-BP for 6 h, then cells were harvested for co-immunoprecipitations by anti-Flag antibody. The inputs were also subjected to immnuoblotting to show the expression levels. Asterisk represents unspecific band.

To assess the effect of 2-BP on TNFα+CHX induced Hela cell death, Annexin V/PI double staining assay was first used (Fig. 6a and Table S3). Between solvent group and 2-BP group, no significant differences were observed in total cell death (Annexin V+ and/or PI+). However, less PI+ cells were detected by flow cytometry and fluorescence microscope in 2-BP group (Fig. 6a–c and Table S3). Because one of the major differences between pyroptosis and apoptosis is the integrity of plasma membrane^9^, the PI+ cells in a short time point might represent pyroptosis better than apoptosis. Thus, 2-BP significantly inhibited TNFα+CHX induced pyroptosis, but not apoptosis. We also found the release of LDH was significantly decreased in the 2-BP treated group compared to control at 9 h and 12 h (Fig. 6d and Supplementary Fig. 9), further supporting the idea that 2-BP could inhibit TNFα+CHX induced pyroptosis.

The predicted palmitoylation sites on GSDME were then mutated to alanine and transfected into Hela cells 24 h followed by TNFα+CHX treatments. A shift of the GSDME-C band was again observed in the WT GSDME transfected cells (Fig. 6e). Moreover, when the potential palmitoylation sites C407/C408 were mutated, the shifted GSDME-C band was diminished (Fig. 6e), providing further evidence that this shifted GSDME-C band was a palmitoylation. However, the shifted band was not abolished, suggesting GSDME may exist other palmitoylation sites or even other posttranslational modifications. Further, compared to the overexpression of WT GSDME, the palmitoylation site mutated GSDME also induced less LDH release at both 3 h and 6 h (Fig. 6f).

C-terminal of GSDMD (GSDMD-C) can still bind to GSDMD-N after cleavage to inhibit its pro-pyroptotic functions^18^. Therefore, 2-BP might inhibit pyroptosis through two possible mechanisms: by inhibiting the GSDME conformation change, which affects the identification and binding between caspase-3 and GSDME; or by inhibiting the modification of GSDME-C, which might facilitate the inhibitory GSDME-C release from GSDME-N after cleavage. Neither 2-BP treatment nor the palmitoylation site mutant inhibited GSDME cleavage (Figs. 5g and 6e), suggesting 2-BP might inhibit pyroptosis through the latter mechanism. To test this, a co-immunoprecipitation assay was performed. As shown in Fig. 6g, in the control group, the interaction between GSDME-N and GSDME-C was barely visible (lane 2). In contrast, interactions between GSDME-N and GSDME-C were observed in both 2-BP pretreated group or in the palmitoylation site mutant group (lane 3 and 4), supporting the idea that palmitoylation facilitated their dissociations.

### Several ZDHHC family proteins interact and palmitoylate GSDME

**Fig. 7:**
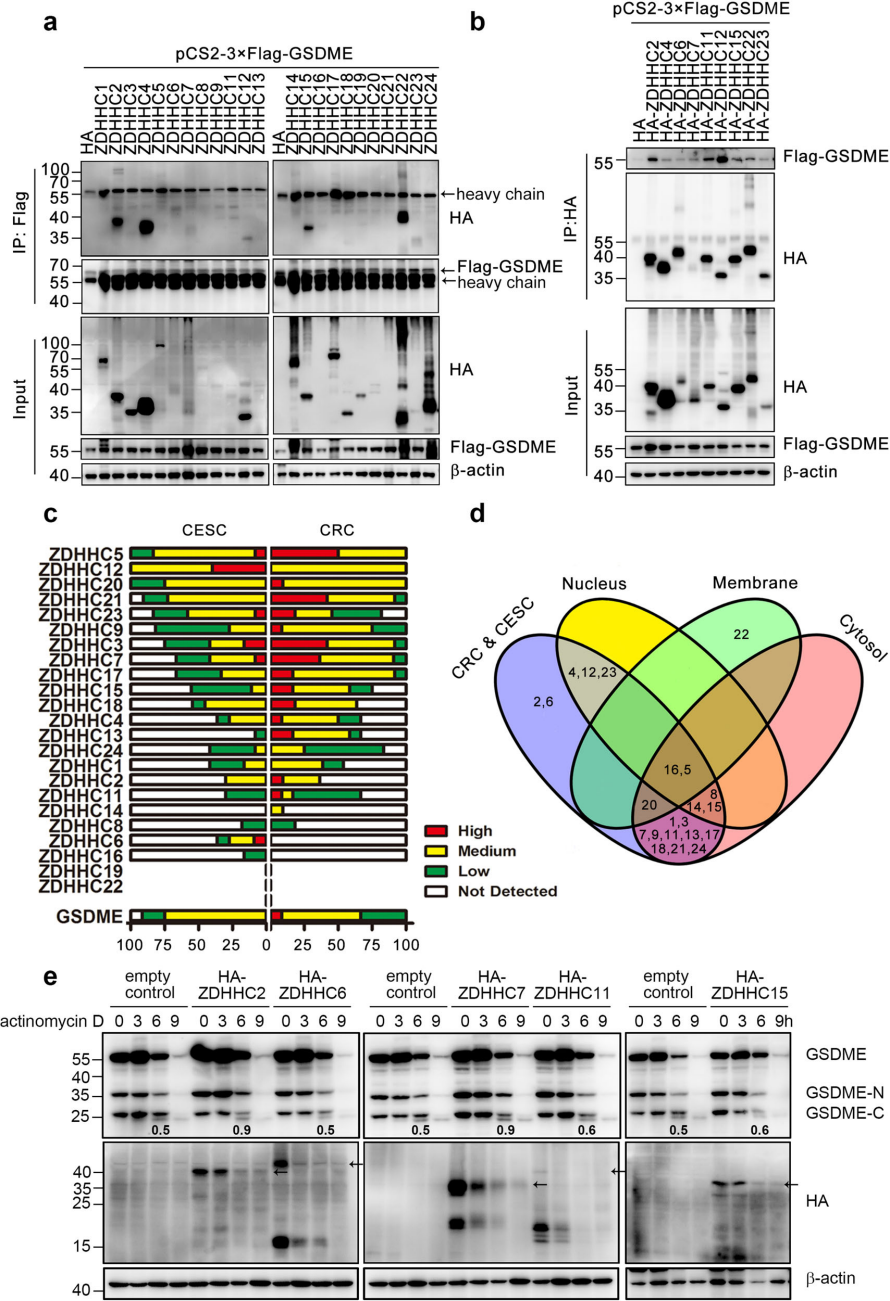
GSDME interacts with ZDHHCs. **a**, **b** After 3×Flag-tagged GSDME and indicated HA-tagged ZDHHCs were co-transfected into 293T cells, co-immunoprecipitations were performed using an anti-Flag antibody (**a**) or an anti-HA antibody (**b**). The inputs were also subjected to immunoblotting to show the expression levels. **c** The expression of GSDME and different ZDHHCs in CESC and CRC were summarized based on the immunohistochemistry results from the Human Protein Atlas. **d** Venn diagram was performed to show the expression patterns of different ZDHHCs in CESC and CRC, including expression level and subcellular distributions. **e** 24 h after Hela cells transfected with the indicated ZDHHC plasmids and then cells were incubated with actinomycin D for the indicated time. The cells were collected for immunoblotting. Numbers indicated the ratio of optical density of shifted GSDME-C band to the unshifted GSDME-C band. Arrows indicated the expression of different ZDHHC proteins.

Palmitoylation is catalyzed by a family of enzymes that share a DHHC domain^44^. There are 23 ZDHHC proteins (ZDHHC1-24, ZDHHC10 is omitted) in humans^44–46^. As shown in Fig. 7a, the co-immunoprecipitation assay indicated ZDHHC 2/4/6/7/11/12/15/22/23 could interact with GSDME. Moreover, a reverse co-immunoprecipitation assay supported the interactions (Fig. 7b). Previous studies have shown ZDHHC proteins have different intracellular localizations^22^. Using the immunohistochemistry results from the Human Protein Atlas (HPA)^47^, ZDHHC-4/12/23 only expressed in nucleus and ZDHHC-22 did not express in both colorectal cancer (CRC) and cervical cancer (CESC), which did not match the subcellular distribution of GSDME (Fig. 7c, d). Thus, ZDHHC-2/6/7/11/15 were potential enzymes to interact with and palmitoylate GSDME.

Hela cells were then transfected with ZDHHC-2/6/7/11/15 respectively, and treated with actinomycin D, which induced a slower palmitoylation of GSDME-C compared to TNFα+CHX (Fig. 5a, b). In contrast to empty vector, after the transfection of ZDHHC-2/7/11/15 but not ZDHHC-6, the shifted band of GSDME-C increased (Fig. 7e), suggesting that GSDME-C could be palmitoylated by ZDHHC-2/7/11/15, and also providing further evidences that the shifted band of GSDME-C was a palmitoylation. Taken together, the results in Fig. 7 suggested ZDHHC-2/7/11/15 could interact with and palmitoylate GSDME-C during chemotherapy treatments.

## Discussion

**Fig. 8:**
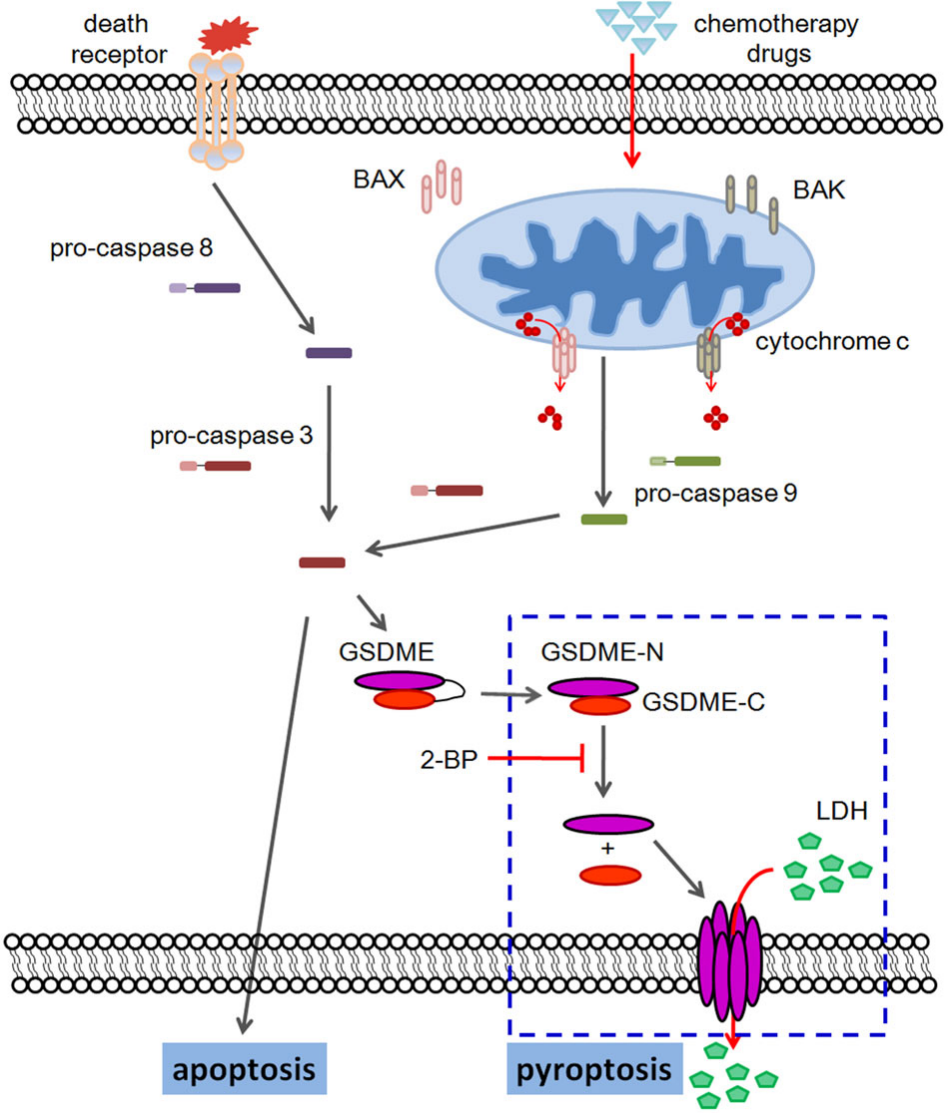
The model of chemotherapy-induced pyroptosis was indicated. MOMP pathway inducers, such as TNFα+CHX, navitoclax and etoposide can activate BAK/BAX to permeabilize the MOM and release cytochrome c into the cytosol. Cytochrome c then activates caspase-9 and subsequent caspase-3, which will cause cell apoptosis or pyroptosis. In pyrototic pathway, GSDME is cleaved by caspase-3 to gnenrate GSDME-N and GSDME-C, where GSDME-N could directly oligomerize and cause plasma membrane lysis. Palmitoylation inhibitors inhibit GSDME-C palmitoylation, therefore inhibit its dissociation from GSDME-N and subsequent pyroptotic characteristics, including LDH release and the formation of plasma membrane bubbles.

In the present study, we found that several chemotherapy drugs induced cancer cell pyroptosis were mediated by the BAK/BAX-caspase-3-GSDME pathway. Furthermore, GSDME was palmitoylated on its C-terminal (GSDME-C) to promote its dissociation from GSDME-N during treatments, resulting in increased chemotherapy drug-induced pyroptosis. The palmitoylation inhibitor 2-BP could therefore inhibit chemotherapy drugs induced pyroptosis. These results suggested a model shown in Fig. 8.

At least three different pathways have been reported to initiate pyroptosis. The first is a pathogen-induced pyroptosis pathway, in which bacteria and microbial infections caused the activation of caspase-1/4/5/11 followed by the cleavage of GSDMD, resulting in the pore formation on the cell plasma membrane^17–19,48^. The second is also initiated by pathogens, where cytosolic LPS causes the caspase-11 dependent cleavage of pannexin-1 followed by the activation of the purinergic receptor P2X ligand-gated ion channel (P2X7), resulting in intracellular ATP release^49^. The third is induced by chemotherapy drugs in cancer cells, where activated caspase-3 induced GSDME cleavage^6,7^. Moreover, the caspase-1-GSDMD mediated pyroptosis pathway is different from the caspase-3-GSDME pathway, because the latter cannot induce IL-1β activation and release^50^.

In this study, we found TNFα+CHX, and navitoclax both induced pyroptotic cell morphologies, LDH release and GSDME cleavage, suggesting pyroptosis was induced by both drugs. Because caspase-3 was activated in this process, we think both pyroptosis and apoptosis were induced. Apoptotic cells theoretically keep the integrity of the cell membrane, thus the PI+ cells might represent pyroptotic cell death more than apoptotic cell death (Fig. 1i, j and Supplementary Fig. 2). On the other hand, due to some other forms of cell death also exhibit PI+, a more specific marker for pyroptotic cell death is still urgently needed.

GSDME knockdown not only diminished the pyroptotic morphology induced by TNFα+CHX and navitoclax, but also diminished the LDH release after both treatments, suggesting the important role of GSDME in the induction of pyroptosis, in agreement with previous studies^7,51,52^. Although GSDME knockdown significantly decreased the LDH release and pyroptotic morphology, however, both were not completely diminished in our study, which might because GSDME was only about half knocked-down.

Upstream of the cleavage of GSDME, chemeotherapy drugs such as cisplatin, topotecan, and doxorubicin have been reported to activate pro-apoptotic caspase-3^7^. In this study, we found that the pan-caspase inhibitor Q-VD-OPh could completely abolish the pyroptosis (Fig. 3), in agreement with the essential role of caspases in pyroptosis^6,7,17–19^. Moreover, when caspase-3 was knocked down or specifically inhibited, the pyroptosis induced also significantly decreased, suggesting that caspase-3 was essential.

The treatments of TNFα+CHX^39^ and navitoclax^53^ have been previously reported to activate MOMP effector proteins BAK and BAX, thereby causing the cytochrome c release. Our results supported the idea that BAK and BAX activation were upstream of GSDME cleavage (Fig. 1 and Supplementary Figs. 1 and 2). Further, our results indicated that either BAK or BAX knockdown could attenuate but not abolish the LDH release, the GSDME cleavage and pyroptotic morphology (Fig. 2 and Supplementary Fig. 6), suggesting that sole BAK and BAX could activate the caspase cascade and subsequent pyroptosis pathways.

Although GSDME have been reported to participate the chemotherapy-induced pyroptosis^6,7^, however, how this process is regulated is not clear. Interestingly, a shift of GSDME-C was observed on the western blotting during TNFα+CHX treatment, suggesting a potential modification. We have tried to harvest this GSDME-C fraction (Supplementary Fig. 8b) for further identification (i.e. Mass Spectrum), however, the immuno-precipitation assay could only harvest the GSDME-N, but not GSDME-C. Previous studies have shown one of the gasdermin family proteins GSDMA could be palmitoylated, thus we speculated whether this band admits the same modification. Firstly, using CSS-Palm 4.0, GSDME was indicated to be palmitoylated at C407/C408. Secondly, when the palmitoylation inhibitor 2-BP or potential palmitoylation site mutations were applied (Figs. 5g and 6e), this shifted band was diminished, suggesting that the modification was a palmitoylation. Finally, some palmitoylation enzymes (ZDHHC proteins) were found to interact with GSDME and induce the GSDME-C band shift (Fig. 7), further supporting the idea that GSDME was palmitoylated by these enzymes. Although these experiments support this modification is a palmitoylation of GSDME-C, a mass spectrum experiment is needed to further confirm this idea in the future.

Palmitoylation is a kind of posttranslational modification that adds saturated 16-carbon palmitic acid to specific cysteine residues, which may influence GSDME-dependent pyroptosis. Posttranslational modifications are found in many other cell death mechanisms, for example, the core necroptotic execution protein MLKL (mixed lineage kinase domain-like protein) was phosphorylated by RIPK3 before its membrane association^54,55^. Therefore, the role of GSDME palmitoylation on drug-induced pyroptosis was investigated. Indeed, this palmitoylation could promote TNFα+CHX induced pyroptosis, which was supported by at least two pieces of evidence. First, cells pre-incubated with palmitoylation inhibitor 2-BP, decreased the LDH release and the PI positive cells after TNFα+CHX treatment (Fig. 6b–d and Supplementary Fig. 9). Second, the predicted palmitoylation site mutant C407A/C408A also released less LDH compared to WT GSDME after TNFα+CHX treatment (Fig. 6f), although this GSDME mutant still induced high percentage of LDH release after TNFα+CHX treatment (Fig. 6f), which might due to the existence of high endogenous GSDME expression (Fig. 1a). Further experiments, such as the usage of GSDME knock-out cells or cells without GSDME expressing might help reduce the effect of endogenous GSDME. On the other hand, we could not rule out the possibility that there may exist other palmitoylation sites or even other kind of posttranslational modifications, because the shifted GSDME-C band and LDH release were not totally inhibited in the mutant group (Fig. 6e, f).

Although we observed several evidences of the transformation of cell death from pyroptosis to apoptosis, such as the PI+ positive cells were decreased while the total cell death were not changed, however, due to the limited probes to discern the two types of cell death, the effect is not big. On the other hand, as previously indicated, TNFα+CHX induced HCT116 cell death includes both pyroptosis and apoptosis, which also limited the phenomenon. Nonetheless, 2-BP transformed part of pyroptotic cells to apoptotic.

After cleavage, the active N-terminal of gasdermin family protein translocates and binds to the lipid bilayers of plasma membrane to induce LDH release and the pyroptotic morphology^56–59^. Moreover, previous study has found the C-terminal of GSDMD can still be an inhibitory of its N-terminal after cleavage, which inhibits its pro-pyroptotic function^18^. We found that both the palmitoylation inhibitors and the palmitoylation site mutant increased the interaction of GSDME-C to GSDME-N (Fig. 6g), providing a potential explanation for the promoted pyroptosis by GSDME-C palmitoylation.

Finally, palmitoylation have shown to be promising targets in the treatment of melanoma^60^ and breast tumor^61^. In our study, we found the palmitoylation inhibitor 2-BP diminished the pyroptosis induced by chemotherapy drugs. Our results indicated 2-BP inhibited chemotherapy-induced pyroptosis (Fig. 6b, c). However, the total cell death were not changed (Fig. 6a), suggesting 2-BP transformed pyroptotic cells to apoptotic. When caspase-3 was activated, cells could either become apoptotic or pyroptotic, depending on GSDME activation (Fig. 8), therefore, the inhibition of pyroptosis downstream of caspase-3 will transform the cells to apoptosis. While pyroptosis was suggested to be involved in the chemotherapy-induced reverse effects such as disruption of the immune system and weight loss in animal studies, our findings offered new targets to achieve the transformation between chemotherapy-induced pyroptosis and apoptosis.

In summary, we identified that BAK and BAX were required for TNFα+CHX and navitoclax-induced pyroptosis and sole BAK or BAX could mediate this process. Moreover, caspase-3 activation and GSMDE cleavage were essential in this pyrotosis pathway. In addition, GSDME-C was found to be palmitoylated by several ZDHHC proteins, which promoted drug-induced pyroptosis. The palmitoylation inhibitor 2-BP could inhibit GSDME-C palmitoylation, and therefore inhibit chemotherapy-induced pyroptosis.

## Materials and methods

### Antibodies and reagents

Antibodies for GSDME (ab225893) and GSDMD (ab209845) were purchased from Abcam. Antibody for β-acin (TA811000) was purchased from OriGene. Antibody for S-tag was kindly gifted by Scott H. Kaufmann (Mayo Clinic, Rochester, MN). Antibody for Flag-tag (F4049) was from Sigma-Aldrich. Other antibodies used in this study include Caspase-1 (2225S), Caspase-3 (9662S), cleaved Caspase-3 (9664S), Caspase-7 (12827S), Caspase-9 (9502S), BAK (12105S), BAX (5023S), BID (8762S), and HA tag were purchased from Cell Signaling Technology (CST). The secondary antibodies including horseradish peroxidase (HRP)-conjugated goat anti-rabbit IgG (7074S) and HRP-conjugated goat anti-mouse IgG (7076S) were from CST.

Reagents were purchased as followings: recombinant human TNFα (rcyc-htnfs) from InvivoGene; cycloheximide (HY-12320), navitoclax (HY-10087), actinomycin D(HY-17559), broad spectrum caspase inhibitor Q-VD-OPh (HY-12305) from MedChemExpress; caspase-3 inhitor Q-DEVD-OPh (1175-1) from Biovision, protease inhibitor cocktail tablets (04693132001) from Roche; protein G sepharose (17-0618-01) from GE Healthcare; Lipofectamine 2000 (11668-019) and Lipofectamine RNAiMAX (13778-150) from Invitrogen; anti-Flag M2 magnetic beads (M8823) and 2-BP (238422) from Sigma-Aldrich; CytoTox 96 Non-Radio cytotoxicity assay kit (G1780) from Promega; Hoechst 33342/PI double stain kit (CA1120) from Solarbio; and FITC annexin V appotosis kit I (556547) from BD Biosciences.

### Cell culture and treatments

The human colon cancer cell lines HCT116 wild type and *BAK*^−/−^*BAX*^−/−^ (DKO), the mouse embryonic fibroblasts MEFs, the human ovarian cancer cell lines including Ovcar3, Ovcar5, Ovcar8, A2780, HeyA8, OV90, COV362, SKOV3, Ovise, and the hematoma cell lines including V937, MOLT-3, MOLT-4, THP-1, SKW6.4, H9, Jurkat, K562, KG1a, RL, ML-1, HL-60, HT, and MV4-11 were kind gifts from Scott H. Kaufmann (Mayo Clinic, Rochester, MN). The mouse macrophage cell lines Raw264.7, Raw264.7-ASC, and the iBMDM (immortalized bone-marrow-derived macrophages) were kindly gifted by Jiahuai Han (Xiamen University, China). The human cervical cancer cell line Hela was gifted by Xin Ye (Institute of Microbiology, Chinese Academy of Sciences). The human embryonic kidney cell line 293T and lung cancer cell line A549 were purchased from CCTCC (China Center for Type Culture Collection). Cell characterization (polymorphic short tandem repeat profiling) and contamination tests were performed. The wild type and DKO HCT116 cells were maintained in McCoy’s 5 A; the 293 T, Hela, MEFs, iBMDM, Raw264.7, and A549 were maintained in DMEM; and all the ovarian cell lines and the lymphohematopoietic cell lines were grown in RPMI-1640. All cells were cultured at 37 °C with 5% CO_2_, supplemented with 10% FBS (fetal bovine serum, FB25015, Clark) and 1% PS (penicillin-streptomycin, SV30010, Hyclone).

For navitoclax or TNFα+CHX treatments, cells were pre-seeded overnight until cell density reached ~60%. The culture medium was switched to fresh medium containing relevant drugs and incubated for a period of time as indicated. To inhibit caspase activity or palmitoylation, cells were pre-incubated for 1 h with Q-VD-OPh or 2-BP, respectively. Unless otherwise specified, the concentrations of the drugs used are: 2 μM for navitoclax, 20 ng/mL for TNFα, 10 μg/mL for CHX, 50 μM for Q-VD-OPh, 50 μM for 2-BP, and 50 μM for actinomycin D.

### Plasmids and transfection

The plasmids encoding HA-tagged ZDHHCs were kindly gifted from Xu Wu (Harvard Medical School, Boston, USA). The plasmid encoding 3× Flag tagged GSDME was kindly gifted by Feng Shao (National Institute of Biological Sciences, Beijing, China). Mutations were generated by site-direct mutagenesis using Fast Mutagenesis Systm (FM111-01) from Transgen. All plasmids were verified by DNA sequencing.

Transient transfection of 293T and Hela cells were performed using Lipofectamin 2000 following the manufacturer’s instructions. Briefly, log phase cells growing in antibiotic-free medium were transiently transfected with the indicated plasmids with a ratio of plasmids: reagent at 1:2 (W/V). After incubation in antibiotic-free medium for 6 h followed by complete medium for another 24 h, cells were treated with drugs or solvent as indicated. At the completion of the incubation, cells were harvested for western blotting and flow cytometry, or the culture supernatants were harvested for the measurement of the percentage of LDH release.

### RNA interference

The siRNA sequences were shown in Table S5. For siRNA knockdown, HCT116 cells were cultured to ~60% confluence at the time of transfection. Transfection of siRNA was performed using the Lipofectamine RNAiMAX according to the manufacturer’s protocol. The transfected cells were grown in antibiotic-free medium for 24 h and then re-seeded for further 24 h before drug treatments. The knockdown efficiency was examined by immunoblotting.

### Microscopy images

Cells were seeded in 6-, 12-, or 24-well plates. After treatments, static bright field images of indicated cells were captured using an Olympus CKX53 at room temperature supplemented with TCapture in blinded experiments. The pictures were processed using ImageJ software. Pyroptotic cells (cells with large bubbles) were counted from 200 random cells under microscopic fields in blinded experiments and the percentage was calculated using the equation pyroptotic cells/total cells × 100% from three independent experiments.

### Hoechst 33342/PI double staining

Cells were seeded in 24-well plates for 24 h and followed by treated as indicated. The cells were then stained with Hoechst 33342 (1 μg/μL) and PI (1 μg/μL) for 30 min at 4 °C. After that, the Leica DMI4000B was used to visualize and take pictures. Three fields per well were randomly selected for counting the stained cells by Image J software.

### LDH release assay

Cells were seeded in 6-, 12-, or 24-well plates and treated as indicated. Culture supernatants were harvested and centrifuged at 300×*g* for 10 min after treatments. Aliquots of supernatants were transferred into 96-well plates, and subjected to the CytoTox 96 assay kit. The percentage of LDH release was calculated using the equation (LDH_sample_ − LDH_background_)/(LDH_maximum_−LDH_background_) × 100%, where LDH_sample_, LDH_background_, and LDH_maximu__m_ are the OD_490_ measured for the drug treated, untreated, and lysis solution (provided in the kit) treated supernatants, respectively. Each sample was tested in triplicates to obtain the average.

### Western blotting

Both cells and culture supernatants were harvested for western blotting. After washing, cell sediments were lysed in RIPA lysis buffer (50 mM Tris, pH 7.4, 150 mM NaCl, 1% Triton X-100, 1% sodium deoxycholate, 0.1% SDS) with cocktail, and sonicated. The total protein concentration was measured by BCA protein assay kit (P0011, Beyotime). Samples were denatured in sample loading buffer (50 mM Tris-HCl, pH 6.8, 2% SDS (W/V), 0.1% BPB (W/V), 10% glycerol (V/V), and 1% β-mercaptoethanol (V/V)). Samples were then separated by SDS-PAGE and transferred to PVDF membranes followed by blocking. The membrane was then incubated overnight with primary antibody against indicated proteins, followed by incubated with HRP-conjugated secondary antibodies. All proteins were visualized with the Tanon High-sig ECL Western Blotting substrate (180-501, Tanon, China). The gray-scale values of GSDME-C and shifted GSDME-C were captured by ImageJ.

### Flow cytometry

Cells were seeded to density about ~60% before drug treatments. Cells were harvested, washed with cold PBS, and stained with the FITC-labeled Annexin V and PI using the FITC Annexin V appotosis kit I. Data was obtained using CytoFLEX (Beckman Coulter) and analyzed by CytExpert software.

### Co-immunoprecipitation

In all, 24 h after transfection, cells were harvested and lysed in lysis buffer (20 mM Tris (pH 7.5), 150 mM NaCl, 1% Triton X-100) containing a protease inhibitor cocktail. In total, 1000 μg of supernatants were incubated with Flag magnetic beads or protein G beads pre-coupled with HA antibody at 4 °C overnight. After washing, beads bound proteins were then released by heating them for 15 min at 100 ºC in sample loading buffer. Samples were subjected to western blotting and probed with the indicated antibodies.

### Statistical analysis

All data was analyzed using GraphPad Prism software. Data was shown as means ± SD. The levels of significance for comparison between samples were determined by Student’s *t*-test. *P* > 0.05 was considered not significant (ns). *P < 0.05, **P < 0.01, ***P < 0.001.

## Supplementary information

Supplementary Figure 1

Supplementary Figure 2

Supplementary Figure 3

Supplementary Figure 4

Supplementary Figure 5

Supplementary Figure 6

Supplementary Figure 7

Supplementary Figure 8

Supplementary Figure 9

Supplementary Figure Legends
