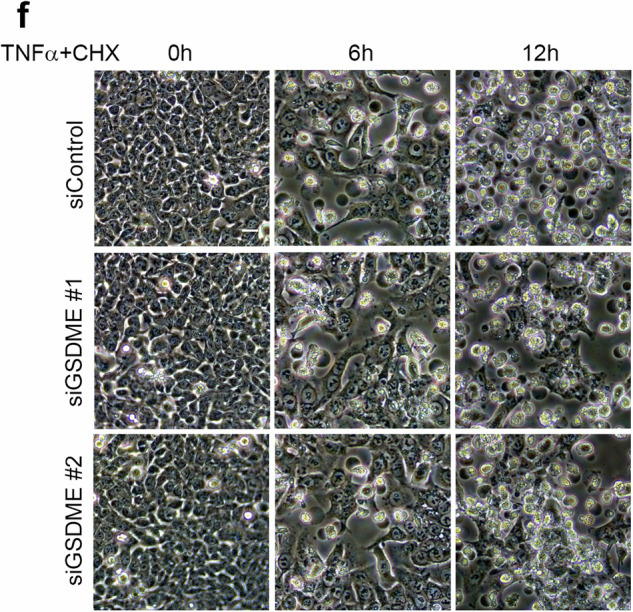# Author Correction: Chemotherapy-induced pyroptosis is mediated by BAK/BAX-caspase-3-GSDME pathway and inhibited by 2-bromopalmitate

**DOI:** 10.1038/s41419-024-07031-8

**Published:** 2024-09-04

**Authors:** Lei Hu, Meng Chen, Xueran Chen, Chenggang Zhao, Zhiyou Fang, Hongzhi Wang, Haiming Dai

**Affiliations:** 1 Anhui Province Key Laboratory of Medical Physics and Technology, Center of Medical Physics and Technology, Hefei Institutes of Physical Science, Chinese Academy of Sciences, 230031 Hefei, China; 2 University of Science and Technology of China, 230026 Hefei, China; 3 Hefei Cancer Hospital, Chinese Academy of Sciences, 230031 Hefei, China

Correction to: *Cell Death and Disease* 10.1038/s41419-020-2476-2, published online 24 April 2020

Due to the large amount of pictures taken during the study, we have made mistakes during the process of combining these pictures together. First, we have put incorrect pictures into Fig. 2d (siBAK #2 6 h and 12 h, and siBAX #1 & siBAK #1 12 h). Second, we have also put incorrect pictures into Fig. 4f (siGSDME #1 6 h).

Now these incorrect pictures have been replaced with the right pictures.

Revised Fig. 2d
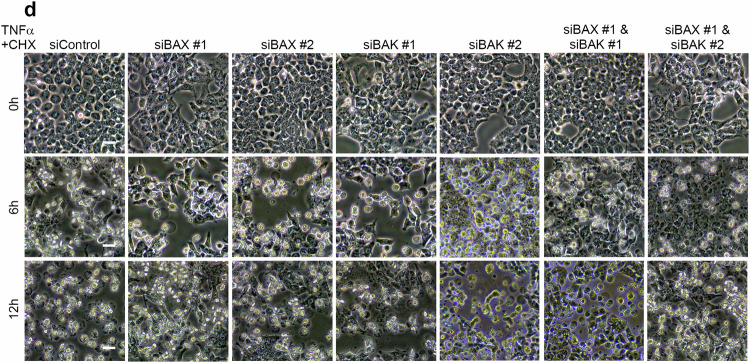


Original Fig. 2d
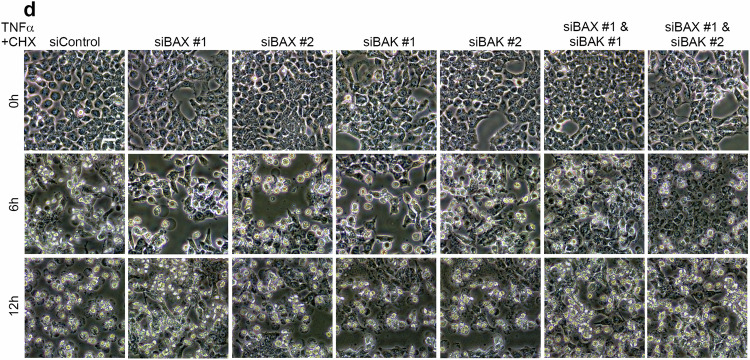


Revised Fig. 4f
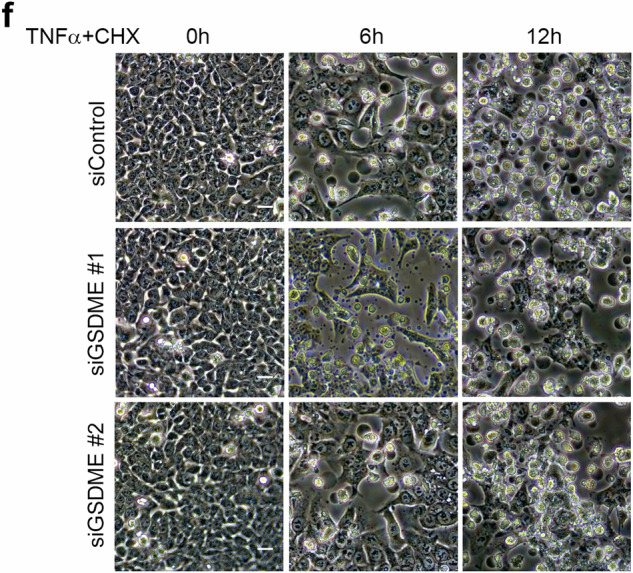


Original Fig. 4f