# Supplementary material for: Chemotherapy-induced pyroptosis is mediated by BAK/BAX-caspase-3-GSDME pathway and inhibited by 2-bromopalmitate
Source: Cell Death Dis. 2020 Apr 24;11(4):281. doi: 10.1038/s41419-020-2476-2 (PMC7181755; doi:10.1038/s41419-020-2476-2)
Supplement: Supplementary file 10 — Supplementary Figure Legends [file 41419_2020_2476_MOESM10_ESM.docx]

**SUPPLEMENTARY FIGURE LEGENDS:**

**Supplementary Fig. 1** BAK/BAX deletion inhibits navitoclax induced pyroptosis. **a** Expresssion levels of GSDMD and GSDME in various cell lines. **b-d** After HCT116 WT and DKO cells were treated with different concentrations of navitoclax for 24 hours, the cells were subjected to immunoblotting analysis (**b**), culture supernatants were collected to measure the percentage of LDH release (**c**), and phase contrast images were captured (**d**).

**Supplementary Fig. 2** A representative experiment for PI and FITC-Annexin V staining. After HCT116 WT and DKO cells were treated with TNFα+CHX (**a**) or navitoclax (**b**) for the indicated time, a representative result was indicated displaying the flow cytometry diagram of PI and FITC-Annexin V staining.

**Supplementary Fig. 3** TNFα+CHX and navitoclax induce Hela cell pyroptosis. After Hela cells were treated with TNFα+CHX (**a, c**) or navitoclax (**b, c**) for the indicated time, culture supernatants were collected to measure the percentage of LDH release (**a, b**) and the time-dependent cell morphorlogies were captured (**c**).

**Supplementary Fig. 4** Actinomycin D and TNFα+CHX induce pyroptosis in Hela and HeyA8 cells respectively. **a-d** Hela (**a, c**) and HeyA8 cells (**b, d**) were treated with actinomycin D and TNFα+CHX respectively for the indicated time. Culture supernatants were collected to measure the percentage of LDH release (**a, b**) and the time-dependent cell morphologies were captured (**c, d**).

**Supplementary Fig. 5** TNFα+CHX and navitoclax induced pyroptosis in HCT116 is BID dependent. **a** The efficiency of BID knockdown was detected by immunoblotting. **b, c** Immunoblotting of GSDME cleavage induced by TNFα+CHX (**b**) or navitoclax (**c**) was performed in BID knockdown HCT116 cells.

**Supplementary Fig. 6** BAK and/or BAX knockdown decreases GSDME cleavage induced by TNFα+CHX or navitoclax. **a, b** 48 hours after HCT116 cells were treated with BAK or/and BAX siRNAs, TNFα+CHX (**a**) or navitoclax (**b**) was added and incubated for the indicated time. The centrifugation sediments were collected for immunoblotting.

**Supplementary Fig. 7** Caspases inhibitors abolish TNFα+CHX or navitoclax- induced HCT116 pyroptosis. **a-d** HCT116 cells were pre-incubated with Q-VD-OPh (**a, b**) or caspase-3 specific inhibitor Q-DEVD-OPh (**c, d**) for 1 hour, then treated by TNFα+CHX (**a, c**) or navitoclax (**b, d**) for the indicated time before phase contrast pictures were captured (**a, b**) or LDH releases were measured (**c, d**).

**Supplementary Fig. 8 GSDME antibody can recognize but not immunoprecipitate GSDME-C. a** 48 hours after Hela cells were transfected with GSDME siRNAs, TNFα+CHX was added and incubated for the indicated time. The centrifugation sediments were collected for immunoblotting. **b** Hela cells were treated with or without TNFα+CHX for 3 hours and then harvested for GSDME immunoprecipitation. Negtive control contained only GSDME antibody.

**Supplementary Fig. 9** 2-BP treatment inhibited TNFα+CHX induced LDH release. LDH release induced by DMSO, 2-BP alone or 2-BP with TNFα+CHX was measured at the indicated time points. Considering 2-BP itself induced LDH release, the inhibition of TNFα+CHX induced LDH release by 2-BP could be more significant if 2-BP itself induced LDH release was subtracted.

**Table S1.** The percentages (%) of single PI positive, single FITC-Annexin V positive and FITC-Annexin V/PI double positive HCT116 (WT or DKO) cells were detected by flow cytometry at the indicated time points after treated with TNFα+CHX or navitoclax. SD, standard deviation, n=3.

| **TNFα+CHX** | | **0h** | | **3h** | | **6h** | | **9h** | | **12h** | |
| --- | --- | --- | --- | --- | --- | --- | --- | --- | --- | --- | --- |
|  |  | **Mean** | **SD** | **Mean** | **SD** | **Mean** | **SD** | **Mean** | **SD** | **Mean** | **SD** |
| **PI^+^/Annexin V^-^** | **WT** | 5.15 | 0.69 | 11.77 | 2.42 | 8.09 | 1.45 | 12.03 | 1.57 | 9.09 | 3.19 |
|  | **DKO** | 2.99 | 1.73 | 2.79 | 1.13 | 4.03 | 2.00 | 4.80 | 1.95 | 3.82 | 1.81 |
| **PI^+^/Annexin V^+^** | **WT** | 5.77 | 0.31 | 5.73 | 1.01 | 5.50 | 0.83 | 5.22 | 0.51 | 5.64 | 1.04 |
|  | **DKO** | 4.64 | 0.53 | 4.34 | 0.82 | 5.30 | 0.57 | 5.55 | 0.43 | 5.48 | 0.13 |
| **PI^-^/Annexin V^+^** | **WT** | 5.32 | 0.27 | 11.68 | 1.01 | 14.09 | 1.68 | 14.74 | 1.67 | 13.07 | 1.49 |
|  | **DKO** | 3.37 | 0.40 | 2.77 | 0.33 | 3.27 | 0.17 | 3.13 | 0.20 | 3.38 | 0.26 |
| **Total** | **WT** | 16.25 | 2.37 | 29.18 | 4.38 | 27.68 | 2.36 | 31.99 | 3.69 | 27.80 | 4.36 |
|  | **DKO** | 11.00 | 3.15 | 9.91 | 2.58 | 12.60 | 3.05 | 13.48 | 2.98 | 12.68 | 2.85 |

| **navitoclax** | | **0h** | | **3h** | | **6h** | | **9h** | | **12h** | |
| --- | --- | --- | --- | --- | --- | --- | --- | --- | --- | --- | --- |
|  |  | **Mean** | **SD** | **Mean** | **SD** | **Mean** | **SD** | **Mean** | **SD** | **Mean** | **SD** |
| **PI^+^/Annexin V^-^** | **WT** | 6.83 | 2.12 | 5.49 | 2.63 | 2.67 | 0.24 | 3.32 | 0.12 | 6.90 | 0.24 |
|  | **DKO** | 3.10 | 0.83 | 1.93 | 0.83 | 0.99 | 0.07 | 1.03 | 0.61 | 0.45 | 0.12 |
| **PI^+^/Annexin V^+^** | **WT** | 6.15 | 1.41 | 3.19 | 0.43 | 1.18 | 0.05 | 3.53 | 0.22 | 6.62 | 0.51 |
|  | **DKO** | 4.31 | 0.22 | 3.52 | 0.35 | 2.78 | 0.82 | 2.24 | 0.45 | 2.55 | 0.48 |
| **PI^-^/Annexin V^+^** | **WT** | 5.07 | 0.70 | 14.63 | 0.69 | 27.29 | 0.68 | 36.76 | 0.89 | 33.12 | 0.61 |
|  | **DKO** | 3.16 | 0.15 | 2.70 | 0.24 | 2.72 | 0.08 | 2.76 | 0.43 | 2.93 | 0.32 |
| **Total** | **WT** | 18.04 | 3.89 | 23.31 | 3.06 | 31.14 | 2.37 | 43.61 | 1.95 | 46.65 | 1.83 |
|  | **DKO** | 10.57 | 1.94 | 8.15 | 1.53 | 6.50 | 2.64 | 6.03 | 1.69 | 5.93 | 2.05 |

**Table S2.** The percentages (%) of single PI positive, single FITC-Annexin V positive, and FITC-Annexin V/PI double positive cells were detected by flow cytometry at the indicated time points after treated with TNFα+CHX or navitoclax in the absence or presence of Q-VD-OPh. SD, standard deviation, n=3.

| **TNFα+CHX** | | **0h** | | | | **6h** | | | | **12h** | | | |
| --- | --- | --- | --- | --- | --- | --- | --- | --- | --- | --- | --- | --- | --- |
| **Q-VD-OPh** | | **-** | | **+** | | **-** | | **+** | | **-** | | **+** | |
|  |  | **Mean** | **SD** | **Mean** | **SD** | **Mean** | **SD** | **Mean** | **SD** | **Mean** | **SD** | **Mean** | **SD** |
| **PI+/Annexin V-** | **WT** | 3.42 | 0.78 | 1.66 | 1.15 | 1.21 | 0.15 | 1.65 | 0.31 | 3.11 | 0.40 | 1.31 | 0.26 |
|  | **DKO** | 0.93 | 0.48 | 0.93 | 0.55 | 0.52 | 0.10 | 0.27 | 0.05 | 0.35 | 0.05 | 0.33 | 0.13 |
| **PI+/Annexin V+** | **WT** | 7.00 | 0.40 | 6.43 | 0.49 | 4.36 | 0.40 | 6.16 | 0.93 | 15.67 | 1.04 | 5.36 | 0.39 |
|  | **DKO** | 5.71 | 0.19 | 5.58 | 0.44 | 3.72 | 0.05 | 5.50 | 0.18 | 3.64 | 0.26 | 4.12 | 0.14 |
| **PI-/Annexin V+** | **WT** | 5.72 | 0.36 | 5.50 | 0.61 | 69.58 | 2.35 | 3.49 | 0.25 | 73.44 | 2.45 | 3.46 | 0.37 |
|  | **DKO** | 3.45 | 0.60 | 2.78 | 0.37 | 4.12 | 0.71 | 2.92 | 0.07 | 3.53 | 0.05 | 2.77 | 0.35 |
| **Total** | **WT** | 16.14 | 0.13 | 13.59 | 0.08 | 75.15 | 2.80 | 11.30 | 0.89 | 92.22 | 1.07 | 10.13 | 0.34 |
|  | **DKO** | 10.09 | 1.07 | 9.29 | 0.98 | 8.36 | 0.76 | 8.69 | 0.21 | 7.52 | 0.22 | 7.22 | 0.34 |

| **navitoclax** | | **0h** | | | | **6h** | | | | **12h** | | | | |
| --- | --- | --- | --- | --- | --- | --- | --- | --- | --- | --- | --- | --- | --- | --- |
| **Q-VD-OPh** | | **-** | | **+** | | **-** | | **+** | | **-** | | **+** | | |
|  |  | **Mean** | **SD** | **Mean** | **SD** | **Mean** | **SD** | **Mean** | **SD** | **Mean** | **SD** | **Mean** | **SD** | |
| **PI+/Annexin V-** | **WT** | 1.49 | 0.07 | 5.74 | 0.92 | 3.52 | 0.90 | 3.66 | 1.41 | 2.46 | 0.60 | 0.71 | | 0.21 |
|  | **DKO** | 0.58 | 0.12 | 1.00 | 0.48 | 0.65 | 0.35 | 0.70 | 0.19 | 0.80 | 0.10 | 0.68 | | 0.05 |
| **PI+/Annexin V+** | **WT** | 5.36 | 0.75 | 6.77 | 0.57 | 6.26 | 0.30 | 8.55 | 0.27 | 7.66 | 0.78 | 6.87 | | 0.87 |
|  | **DKO** | 4.93 | 0.97 | 6.28 | 0.75 | 4.67 | 0.44 | 7.42 | 0.89 | 7.08 | 0.70 | 9.29 | | 0.82 |
| **PI-/Annexin V+** | **WT** | 7.05 | 0.37 | 2.92 | 0.25 | 27.58 | 2.07 | 5.12 | 0.53 | 33.56 | 1.25 | 9.22 | | 0.86 |
|  | **DKO** | 2.99 | 0.41 | 3.18 | 0.25 | 2.85 | 0.45 | 3.10 | 0.64 | 3.22 | 0.42 | 4.55 | | 0.92 |
| **Total** | **WT** | 13.90 | 1.05 | 15.43 | 0.14 | 37.35 | 1.35 | 17.33 | 0.68 | 43.68 | 1.65 | 16.81 | | 1.89 |
|  | **DKO** | 8.50 | 1.44 | 10.46 | 1.13 | 8.17 | 1.15 | 11.22 | 1.37 | 11.10 | 1.04 | 14.52 | | 1.71 |

**Table S3.** The percentages (%) of single PI positive, single FITC-Annexin V positive, and FITC-Annexin V/PI double positive cells were detected by flow cytometry at the indicated time points after treated with TNFα+CHX in the presence of DMSO or 2-BP. SD, standard deviation, n=3.

| **TNFα+CHX** | | **0h** | | **3h** | | **6h** | | **9h** | | **12h** | |
| --- | --- | --- | --- | --- | --- | --- | --- | --- | --- | --- | --- |
|  |  | **Mean** | **SD** | **Mean** | **SD** | **Mean** | **SD** | **Mean** | **SD** | **Mean** | **SD** |
| **PI^+^/Annexin V^-^** | **DMSO** | 5.28 | 1.82 | 13.66 | 1.48 | 29.87 | 0.99 | 34.76 | 0.60 | 12.36 | 0.10 |
|  | **2-BP** | 5.05 | 0.41 | 10.76 | 1.09 | 19.70 | 0.51 | 33.99 | 079 | 9.29 | 0.15 |
| **PI^+^/Annexin V^+^** | **DMSO** | 6.89 | 2.31 | 12.14 | 0.47 | 16.71 | 0.61 | 26.01 | 0.57 | 53.41 | 0.19 |
|  | **2-BP** | 9.00 | 0.07 | 11.07 | 0.39 | 25.79 | 0.28 | 27.71 | 0.77 | 38.02 | 0.64 |
| **PI^-^/Annexin V^+^** | **DMSO** | 2.34 | 0.04 | 4.96 | 0.64 | 9.35 | 0.43 | 11.06 | 0.68 | 20.98 | 0.28 |
|  | **2-BP** | 2.25 | 0.03 | 13.19 | 0.06 | 14.15 | 0.16 | 13.85 | 0.21 | 38.60 | 033 |
| **Total** | **DMSO** | 14.51 | 3.10 | 30.76 | 2.75 | 55.93 | 2.57 | 71.83 | 1.66 | 86.75 | 1.26 |
|  | **2-BP** | 16.30 | 2.48 | 35.02 | 1.89 | 59.64 | 1.31 | 75.55 | 2.39 | 85.91 | 0.18 |

**Table S4. Palmitoylation sites predicted by CSS-Palm 4.0.**

| **Protein** | **Palmitoylation Site^a^ (Cysteine)** | **Score** | **Cutoff** | **Threshold** |
| --- | --- | --- | --- | --- |
| GSDME | 407 | 5.521 | 3.419 | High |
|  | 408 | 6.294 | 4.222 |  |
| CD9 | 9 | 21.47 | 13.877 |  |
|  | 218 | 16.664 | 3.419 |  |
|  | 219 | 5.184 | 4.222 |  |
| CLIP3 | 534 | 10.507 | 3.419 |  |
|  | 535 | 6.163 | 4.222 |  |
| HRAS | 181 | 15.98 | 13.877 |  |
|  | 184 | 16.844 | 13.877 |  |
| RHOB | 189 | 15.102 | 13.877 |  |
|  | 198 | 13.675 | 3.419 |  |
| GRK6 | 561 | 17.745 | 3.419 |  |
|  | 562 | 5.86 | 4.222 |  |
| GAP43 | 3 | 17.85 | 3.419 |  |
|  | 4 | 45.561 | 4.222 |  |
| CKAP4 | 100 | 12.396 | 10.722 | Medium |
| NRAS | 181 | 13.029 | 10.722 |  |
| RGS4 | 99 | 10.825 | 10.722 |  |
|  | 109 | 11.683 | 10.722 |  |

a: All palmitoylation sites except GSDME have been reported.

**Table S5. siRNA used to knockdown target mRNA**

| **Primer** | **Primer sequence (5’ to 3’)** |
| --- | --- |
| siControl | TTCTCCGAACGUGUCACGUTT |
| siGSDME #1 | GATGATGGAGTATCTGATCTT |
| siGSDME #2 | GCGGTCCTATTTGATGATGAA |
| siBid #1 | GGGATGAGTGCATCACAAATT |
| siBid #2 | CCTTGCTCCGTGATGTCTTTT |
| siBak #1 | GTACGAAGATTCTTCAAAT |
| siBak #2 | CCCATTCACTACAGGTGAA |
| siBax #1 | GACGAACTGGACAGTAACA |
| siBax #2 | TATGGAGCTGCAGAGGATG |
| siCaspase3 #1 | GCAGCAAACCTCAGGGAAATT |
| siCaspase3 #2 | GGAATATCCCTGGACAACAGTTATA |
